# Normative data for idiomatic expressions

**DOI:** 10.3758/s13428-016-0705-5

**Published:** 2016-02-23

**Authors:** Emily Nordmann, Antonia A. Jambazova

**Affiliations:** School of Psychology, William Guild Building, University of Aberdeen, Aberdeen, AB24 2UB UK

**Keywords:** Idioms, Normative data, Novel idioms, Familiarity, Literality, Decomposability, Meta-analysis

## Abstract

Idiomatic expressions such as *kick the bucket* or *go down a storm* can differ on a number of internal features, such as familiarity, meaning, literality, and decomposability, and these types of features have been the focus of a number of normative studies. In this article, we provide normative data for a set of Bulgarian idioms and their English translations, and by doing so replicate in a Slavic language the relationships between the ratings previously found in Romance and Germanic languages. Additionally, we compared whether collecting these types of ratings in between-subjects or within-subjects designs affects the data and the conclusions drawn, and found no evidence that design type affects the final outcome. Finally, we present the results of a meta-analysis that summarizes the relationships found across the literature. As in many previous individual studies, we found that familiarity correlates with a number of other features; however, such studies have shown conflicting results concerning literality and decomposability ratings. The meta-analysis revealed reliable relationships of decomposability with a number of other measures, such as familiarity, meaning, and predictability. Conversely, literality was shown to have little to no relationship with any of the other subjective ratings. The implications for these relationships in the context of the wider experimental literature are discussed, with a particular focus on the importance of attaining familiarity ratings for each sample of participants in experimental work.

Idioms are phrases that are more than the sum of their parts. Although the spectrum is broad and varied, the phrases most classically considered idioms are those in which the figurative meaning is unrelated to any literal meaning the phrase may carry (e.g., *kick the bucket* figuratively means *to die suddenly* but literally means *to strike a pail with one’s foot*). This duality of meaning is moderated by varying degrees of compositionality (e.g., some idioms, like *jump the gun* [*act before the appropriate time*], have a closer relationship between their literal and figurative meanings) and raises interesting questions for researchers concerned with studying language. Idioms differ in their numbers of internal features, and although not as comprehensive as the resources that exist for single words, a number of databases provide normative data for idiomatic expressions in English (e.g., Cronk, Lima, & Schweigert, 1993; Libben & Titone, 2008; Nordmann, Cleland, & Bull, 2014; Titone & Connine, 1994), French (Bonin, Méot, & Bugaiska, 2013; Caillies, 2009), and Italian (Tabossi, Arduino, & Fanari, 2011).

Given that the focus of the present article is the relationships between these features, it is worthwhile specifying our working definitions of each, since a number of variations have been used in the literature.

Although *familiarity* is most commonly defined (Libben & Titone, 2008; Tabossi et al., 2011; Titone & Connine, 1994) as the frequency with which a listener or reader encounters a phrase in its spoken or written form (Gernsbacher, 1984), the instructions for assessing participants’ familiarity with idiomatic expressions vary across studies. Bonin et al. (2013) and Tabossi et al. (2011) asked participants to rate how well they thought the idiom was known by other people like them regardless of their personal familiarity with the phrase. Additionally, Bonin et al. assessed subjective frequency—the frequency with which a participant thinks he or she has read, heard, or produced an idiom. For the purposes of this article, we adopt the Gernsbacher definition of familiarity and use instructions adapted from Titone and Connine (1994), which were also used in Libben and Titone (2008) and Nordmann et al. (2014): That is, “decide how frequently you have seen, heard, or used the idiom without taking into account if you know what it means.” Importantly, participants are asked to rate familiarity independently of their personal knowledge of its *meaning*, the second feature of interest.

The fixed nature and prevalence of idioms means that speakers can know the form of an idiom without necessarily knowing the meaning, a phenomenon that can sometimes amusingly manifest in the shape of “eggcorns” (Ching, 2008), or erroneous reshapings of fixed expressions based on phonology (*for all intensive purposes*, *she was an escape goat*). It is therefore important to separate the constructs of meaning and familiarity, because although the two are strongly correlated (Nordmann et al., 2014), they are not synonymous. Meaning is most often measured by asking participants to rate how well they know what an idiom means by considering whether they could use it themselves (e.g., Nordmann et al., 2014; Titone & Connine, 1994), although Tabossi et al. (2011) required participants to explain the meaning, which was then scored by the researchers as correct or incorrect. *Knowledge* (Tabossi et al., 2011) and *meaningfulness* (Libben & Titone, 2008) are also sometimes used synonymously. However, Jolsvai, McCauley, and Christiansen (2013) contrasted the “meaningfulness” of idioms with nonmeaningful fragments, and therefore we adopt the term and operationalization of *meaning* from Titone and Connine to avoid confusion.

*Literality* concerns whether the idiom has a plausible literal interpretation, which is measured across the literature by asking participants to rate whether the phrase could be used literally in addition to its figurative meaning (Caillies, 2009; Libben & Titone, 2008; Nordmann et al., 2014; Tabossi et al., 2011; Titone & Connine, 1994). *Decomposability* (whether the literal meanings of the component words of the idiom contribute to its figurative meaning) and *transparency* (closely related to decomposability, and often used synonymously; the degree to which an idiom’s figurative meaning can be ascertained from its component words) are measured by asking participants to consider the contributions of the component words to the overall figurative meaning. Some studies (Libben & Titone, 2008; Titone & Connine, 1994) ask participants to sort idioms categorically (decomposable/nondecomposable), whereas others (Bonin et al., 2013; Caillies, 2009; Nordmann et al., 2014; Tabossi et al., 2011) collect ratings using Likert scales. Nordmann et al. (2014) argued that continuous scales are preferable to categorical scales, so for the present article we measured decomposability with a Likert scale. *Predictability* refers to the likelihood of a phrase being completed idiomatically, and has previously been measured using cloze tasks (e.g., Bonin et al., 2013). Finally, *syntactic flexibility* (whether the idiom’s syntax can be altered and still retain its figurative meaning) has been assessed by presenting participants with idioms subjected to syntactic modifications (e.g., adverb insertion) and asking them to rate how similar they are in meaning to the original, unmodified phrase, on a Likert scale (Tabossi et al., 2011).

Typically, experimental work seeks either to manipulate or control for these features. For example, Cacciari and Tabossi (1988) used idioms that were low in predictability and could be completed literally until the last word (e.g., *the tennis player was in seventh heaven/place*) to investigate the time-courses of literal and figurative activation. In addition, a number of normative studies have investigated the relationships between these ratings (see Nordmann et al., 2014, for a review), and some have also provided objective measures of frequency for the component words (Bonin et al., 2013; Libben & Titone, 2008). Strong, stable relationships have been found between a number of the features, such as the positive correlations between familiarity and both meaning (Bonin et al., 2013; Libben & Titone, 2008; Nordmann et al., 2014; Tabossi et al., 2011; Titone & Connine, 1994) and predictability (Bonin et al., 2013; Libben & Titone, 2008; Tabossi et al., 2011; Titone & Connine, 1994). For other ratings, a pattern is beginning to emerge. For example, several studies have shown a positive relationship between familiarity and decomposability (Bonin et al., 2013; Libben & Titone, 2008; Nordmann et al., 2014), with highly decomposable idioms such as *make up your mind* (*make a decision*) being rated as more familiar than low-decomposability idioms such as *go down a storm* (*be enthusiastically received by an audience*), although this is not as consistent as the correlation between meaning and predictability. Finally, some relationships are still unclear, particularly those concerning literality, for which contradictory results have been found. For example, whereas Nordmann et al. (2014) found a positive correlation between familiarity and literality, the only other study to show a significant correlation was Tabossi et al. (2011), and this relationship was in the opposite direction.

One issue with studying the effects of familiarity on idiom processing is that it can be difficult to select idioms that are high or low in familiarity for all participants. Nordmann et al. (2014) studied the reliability of ratings given by native and nonnative speakers and found extremely low reliability for all measures, including familiarity (Krippendorff’s alpha = .27). Their results also showed that differences between native and nonnative ratings of literality and decomposability were attributable to familiarity, highlighting the importance of the measure. Nordmann et al. argued that although the relationships between the ratings may be reliable, this should not be confused with interrater reliability. If Participant A rates Idiom 1 as high in familiarity, it is likely that this participant will also rate it as high in meaning; there is no guarantee, however, that Participant B will also rate Idiom 1 as highly familiar.

A method in previous research has been to use novel idioms that have been translated from other languages as the low-familiarity items (e.g., Cain, Oakhill & Lemmon, 2005; Cain, Towse & Knight, 2009). The advantage of this method is that the phrases are natural idioms, and therefore contain all of the features and idiosyncrasies of natural idioms, rather than being artificially created by a researcher. The advantage of translated novel idioms is that one can be certain that they will be extremely low in familiarity to participants, assuming that participants have no knowledge of the language from which they were translated. This means that although there may still be variance in how familiar the high-familiarity items are to individual participants, the contrast between the high- and low-familiarity items should be strong enough for experimental manipulation. Researchers should be aware that translated idioms may not always be suitable, since idiomatic expressions can be culturally specific and influenced by the popularity of a particular theme within a culture. For example, American English generates a large number of baseball-related idiomatic expressions (e.g., *first base*), whereas in British English a range of idioms are produced from the domain of horse racing (e.g., *dark horse*; Boers, Demecheleer, & Eyckmans, 2004). Therefore, it is important to check whether translated idioms are recognizable as such, due to unfamiliar cultural themes. A methodological disadvantage of using translated idioms is that none of the existing databases that provide normative data currently provide translations of the idioms and their meanings into another language. Additionally, none of the studies that have used translated idioms provide normative data from native speakers of the original language. It would be beneficial to have a resource that provided normative data for idioms in their original language and for the L2 translations, so that translated items could still be chosen according to, for instance, literality.

The first aim of this article was to provide normative data for a set of Bulgarian idioms with English translations of the idioms and their meanings. By gathering these data, we can also determine whether the relationships observed in previous research are replicated with idioms from a Slavic language, which would allow us to broaden our generalizations beyond Germanic and Romance languages.

The second aim of this article was to empirically test the effect of the experimental design that is used to collect ratings. Nordmann et al. (2014) used a within-subjects design—that is, all participants rated all idioms on familiarity, meaning, literality, and decomposability, and all participants conducted the ratings in that order. Nordmann et al. argued that, because familiarity appears to have a large effect on processing and is correlated with many other types of ratings, it is important to collect these ratings within subjects, because they can never be independent and should not be treated as such. Correlating, for example, familiarity and literality ratings from independent groups of participants may distort the conclusions drawn.

However, other groups of researchers (e.g., Bonin et al., 2013; Caillies, 2009; Cronk et al., 1993; Tabossi et al., 2011) have used a between-subjects design or a mix of the two (Libben & Titone, 2008; Titone & Connine, 1994). The argument for the use of a between-subjects design is that the ratings of one feature may be influenced by the participant having just rated the idioms on another feature.^1^ A between-subjects design therefore removes the possibility of order effects. In a within-subjects design, participants must always rate familiarity and meaning before they rate decomposability (since the decomposability ratings provide the definition of the idiom). Although we believe that the advantages of using a within-subjects design outweigh the advantages of using a between-subjects design, we cannot, and do not, dismiss the validity of these concerns. In Study 2, we sought to test these conflicting opinions empirically. One-hundred British English idioms were rated in a within-subjects design on familiarity, meaning, literality, and decomposability. The same set of idioms were also rated in a between-subjects design to determine whether there were differences in (a) the mean ratings for each feature and (b) the patterns of correlations between the ratings.

Finally, given the increasing number of studies that have measured relationships between idiom characteristics, we felt that it would be useful to conduct a meta-analysis on the reported relationships. Although consistent relationships have been found across the literature between, for instance, familiarity and meaning, other relationships are more controversial. Nordmann et al. (2014) found that high-literality idioms like *roll up your sleeves* (*prepare to fight or work*) were rated as more familiar than low-literality idioms such as *pay through the nose* (*pay much more than a fair price*), whereas Tabossi et al. (2011) found the opposite pattern. The relationships between the features are likely to be sensitive to the particular idioms used in each study, and therefore a meta-analysis can provide a broader view of the relationships and might allow us to begin to draw generalizable conclusions across items and languages.

## Study 1

### Method

#### Participants

Two-hundred seventeen native Bulgarian participants were recruited from Bulgarian online social media groups (e.g., Aberdeen University Bulgarian Society, Bulgarian Society UK) and Bulgarian language forums. The time taken to complete the study was recorded and participants who had taken less than 20 min to complete the survey were dropped from the analysis as pilot testing indicated it was not possible to complete the study legitimately in less time. In addition, participants who failed to complete an entire section of the survey (e.g., all decomposability ratings) were removed from the analysis. A total of 57 participants were excluded using these criteria, leaving a total of 160 participants (77 female, 83 male; mean age = 31.2, *SD* = 9.66, range = 18–60).

In addition, 43 native British English speakers were recruited using social media and research mailing lists. Participants were asked whether they had any knowledge of Bulgarian, and one participant was excluded for this reason. Using the same exclusion criteria as for the Bulgarian participants, a further six participants were removed from the analyses, leaving a total of 36 participants (28 female, eight male; mean age = 34.47, *SD* = 11.17, range = 18–60).

#### Materials and design

A total of 90 Bulgarian idioms were selected from Banova and Dimova (2014) and an online dictionary (*Bulgarian Phrases and Expressions*, 2014). We selected the idioms so as to have what we judged to be a range of literality scores, but other than that the items were chosen randomly, and therefore differed in syntactic structure and length. This follows Titone and Connine (1994) and Nordmann et al. (2014) and was done to accommodate the use of the items in a range of potential research designs and stimulus selection criteria. The instructions for each rating scale were those used by Nordmann et al.

The idioms and their meanings were then translated by a native Bulgarian–English bilingual speaker (the second author) and checked by a native British English speaker (the first author) to ensure that the idioms were not also present in English. Any idiom that was judged to have an English equivalent was excluded. For example, Като слон в стъкларски магазин (*like an elephant in a glassware shop*) is very similar, both literally and figuratively, to the English idiom *like a bull in a china shop* (meaning = *clumsy*), and so was removed from the final set. The idioms were translated word for word into English, and their syntactic structures were rendered into correspondent English structures without changing the literal meaning. The translated meaning of polysemous words was dependent upon contextual factors. For example, in Настъпвам лъва по опашката (*I step on the lion’s tail*), the noun опашка has two possible meanings (*queue* and *tail*). The translated meaning of the noun опашка was chosen according to the intended meaning of the noun within the context of the idiom (i.e., *tail*).

The translations of the instructions, idioms, and their meanings were verified and edited by an independent Bulgarian–English bilingual speaker and then refined by the second author on the basis of the provided revisions. The Bulgarian participants were given all materials in Bulgarian, and the British English speakers were given all materials in English. The materials and results, including both the Bulgarian and English translations, are freely available at http://osf.io/igqyn.

#### Procedure

Participants first gave their consent and then were given general instructions about the task. Each participant then rated all idioms on familiarity, meaning, literality, and decomposability on a 7-point Likert scale. Before each type of rating, full instructions with examples were given. All participants completed the types of ratings in the same order, and the idioms were presented in the same fixed random order to all participants. The decomposability ratings provided the idiom and its meaning, and for this reason decomposability was presented as the final rating, so that the meaning could not influence judgments about, for instance, literality.

### Results

The normative data are available to download at http://osf.io/igqyn. Table 1 provides a summary of the ratings. Only two translated idioms had a mean familiarity rating above 2 when rated by English speakers—*I caught the devil by its tail* (*M* = 2.25) and *A big stick* (*M* = 2.64)—suggesting that we were successful in our attempt to only provide idioms that did not have close literal or figurative translations in either language.

**Table 1:** Mean ratings and *SD* for each measure by language

	Bulgarian Mean (*SD*)	English Mean (*SD*)
Familiarity	5.26 (1.66)	1.18 (.46)
Meaning	6.37 (1.23)	2.41 (1.56)
Literality	3.73 (2.18)	3.78 (1.92)
Decomposability	3.18 (2.13)	2.54 (1.56)
Length (words)	3.82 (1.14)	5.49 (1.40)

#### Reliability

Reliability analyses were conducted to determine whether the same pattern of low interrater reliability found by Nordmann et al. (2014) for English idioms would be replicated. Krippendorff’s alpha (Hayes & Krippendorff, 2007) was calculated using the “irr” package (Gamer, Lemon, Fellows, & Singh, 2012) using R (R Development Core Team, 2012). Replicating the findings of Nordmann et al., reliability was low for all types of ratings for both types of speakers (see Table 2). It is also worth noting that the larger Bulgarian sample (*n* = 160 vs. *n* = 36) did not appear to improve reliability. The coefficients from both samples for all measures fell between .1 and .4, with Krippendorff (1980) suggesting that coefficients of <.67 as unacceptable.

**Table 2:** Krippendorff’s alpha for each rating type by participant type

	Familiarity	Meaning	Literality	Decomposability
Bulgarian	.193	.188	.226	.124
English	.126	.203	.385	.297

#### Correlations between the ratings

**Fig. 1:**
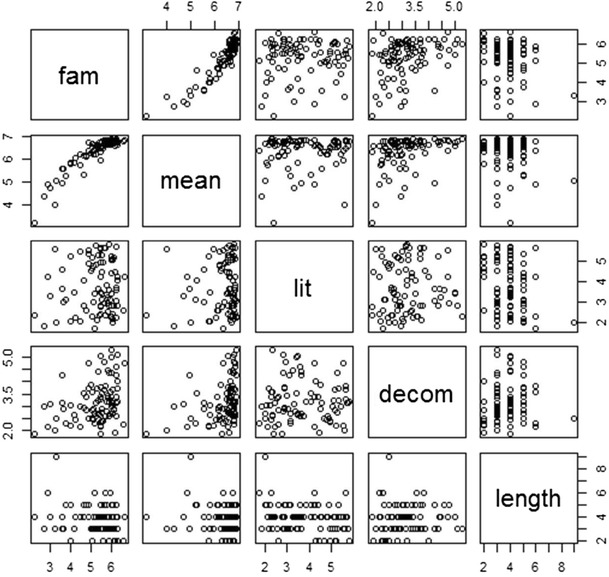
Scatterplot matrix for Bulgarian ratings

**Fig. 2:**
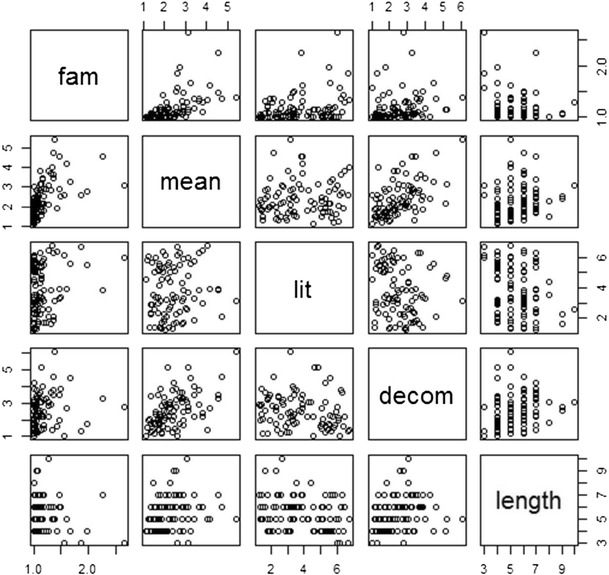
Scatterplot matrix for English ratings

Following Libben and Titone (2008) and Nordmann et al. (2014), Spearman’s rank order correlations were performed between the mean ratings for each item on all of the rating measures: familiarity, meaning, literality, and decomposability (see Table 3 and Figs. 1 and 2). In addition, idiom length was entered as an objective measure; however, due to a lack of information regarding frequency in Bulgarian, noun frequency and verb frequency were not included as in Bonin et al. (2013) and Libben and Titone. Following Tabossi et al. (2011), to control for multiple correlations, alpha was set at *p* < .01.

**Table 3:** Spearman’s correlations between the ratings for Bulgarian and translated English idioms

Language	Meaning		Literality		Decomposability		Length	
	Bul.	Eng.	Bul.	Eng.	Bul.	Eng.	Bul.	Eng.
Familiarity	.85^**^	.69^**^	.01	.22	.33^*^	.31^*^	–.12	–.07
Meaning			–.03	.11	.42^**^	.51^**^	–.03	.01
Literality					.06	–.22	–.19	–.13
Decomposability							.13	.04

^*^ *p* < .01, ^**^ *p* < .001

The significant correlations between familiarity and meaning replicate the findings from all previous normative studies. In addition, the significant positive correlation between familiarity/meaning and decomposability replicates the results of Bonin et al. (2013), Libben and Titone (2008), Nordmann et al. (2014), and Titone and Connine (1994), suggesting that this relationship is stable across items and languages. We failed to replicate the positive relationship found by Nordmann et al. between familiarity and literality and between literality and decomposability. Given the lack of reliability of this effect across studies and languages, it is possible that these relationships are item-specific rather than generalizable to idioms as a whole, an issue that is dealt with in the meta-analysis in Study 3.

#### Effects of participant age on ratings

Our sample included an age span of 18–60 years, and the relatively large number of participants in the Bulgarian group provided us with an opportunity to conduct post-hoc analyses on the effect of age upon the ratings. We constructed linear mixed-effects models using the lme4 package (Bates, Mächler, Bolker, & Walker, 2015) using R (R Development Core Team, 2012). All models included age as a fixed effect, as well as participants and items as random effects. Two models (familiarity and meaning) included a random by-item slope for age (this slope was removed in the models with literality and decomposability, to allow convergence). The age variable was centered.

Age was a significant predictor of meaning and literality ratings, and was marginally significant for familiarity (see Table 4), with older participants being more likely to rate the idioms as more familiar, more meaningful, and more literal than younger participants. For decomposability, age was not a significant predictor.

**Table 4:** Results of linear mixed-effect models for age analyses

	*β*	*t*	*p*
Familiarity	.018	1.92	.055
Meaning	.025	3.66	<.000
Literality	.020	2.36	.018
Decomposability	.003	0.22	.826

The age analyses were post-hoc, and although our sample contained a broad range, age was still positively skewed (skewness = .74). We present the results of this analysis in order to draw attention to this potential source of variance in idiom ratings. It is possible that age differences are the source of the low levels of reliability found, and age differences in the samples across studies may explain some of the conflicting findings. Age differences in idiom processing have been investigated in experimental work; for example, Westbury and Titone (2011) found that older adults were more sensitive to conflicts between the literal and figurative meanings of an idiom. However, as yet there is no robust evidence for how age impacts normative data, and we suggest this as an avenue for future research, given our exploratory findings.

With a previous version of this article, a reviewer highlighted the issues discussed in the introduction regarding the use of a within-subjects design used to collect the data for Study 1. In Study 2 we therefore sought to test the effect that between- and within-subjects designs have on ratings.

## Study 2

### Method

#### Participants

In total, 152 British English speakers participated. Fourteen of the participants were undergraduate psychology students at the University of Aberdeen and participated for course credit. One-hundred thirty-eight participants were recruited online using social media. Thirty-two of these participants took part in the within-subjects ratings and rated all idioms on familiarity, meaning, literality, and decomposability, in that order. Following the same exclusion criteria as in Study 1, two participants were removed from the analysis. The other 120 participants took part in the between-subjects condition, with 30 participants each rating the idioms on either familiarity, meaning, literality, or decomposability (see Table 5).

**Table 5:** Participant information for each group

	Females	Age (*SD*)	Age Range	Age Missing
Within-subjects	19	36.44 (11.55)	19–60	5
Fam-only	18	36.38 (10.17)	18–60	4
Mean-only	19	37.57 (13.38)	18–60	2
Lit-only	22	35.81 (11.42)	18–52	9
Decom-only	17	39.95 (15.69)	18–60	8

*N* = 30 for each group.

#### Materials

One-hundred British English idioms were selected from the *Oxford English Dictionary of Idioms* (Ayto, 2010) and were controlled for length (four words) and syntactic structure: VP [V + PP (P + NP)]—for example, *cut to the chase*, *come to your senses*—in order to more closely match the stimuli used in previous between-subjects studies (e.g., Bonin et al., 2013). The idioms were randomized and presented to all participants in the same random order for each type of rating.

#### Procedure

Participants were asked to verify that they were native British English speakers, and if not, to specify their first language. For the purposes of this article, the data from nonnative speakers are not considered. Participants were randomly assigned to one of the five conditions (all ratings, familiarity only, meaning only, literality only, or decomposability only) on the basis of the date of their birthday. Participants in the within-subjects condition rated all 100 idioms on familiarity, meaning, literality, and decomposability, in that order. The same scale and instructions were used as in Study 1.

### Results

Full normative data are available to download at http://osf.io/igqyn. Table 6 provides a summary of the ratings.

**Table 6:** Mean ratings and *SD* for each measure by design type

	Within-Subjects Mean (*SD*)	Between-Subjects Mean (*SD*)	Combined Mean (*SD*)
Familiarity	5.17 (1.40)	4.76 (1.59)	4.99 (1.53)
Meaning	6.27 (1.10)	6.26 (1.20)	6.28 (1.18)
Literality	4.81 (1.85)	4.56 (1.89)	4.69 (1.90)
Decomposability	4.05 (1.80)	4.12 (1.59)	4.10 (1.72)

#### Reliability

Following the same procedure as in Study 1, Krippendorff’s alpha was calculated for each type of rating in each design type. Again, reliability was low for all ratings and was comparable when the between- and within-subjects ratings were combined (see Table 7). The results mirror those found by Nordmann et al. (2014), that the interrater reliability for normative idiom data is extremely poor, highlighting the importance of collecting participant-specific ratings in experimental work.

**Table 7:** Krippendorff’s alpha for each rating type by design type

	Familiarity	Meaning	Literality	Decomposability
Within-subjects	.310	.251	.299	.260
Between-subjects	.331	.174	.293	.313
Combined	.332	.217	.299	.283

#### Comparison of the mean ratings by design type

The mean ratings for familiarity, meaning, literality, and decomposability by design type (between-/within-subjects) were compared using a one-way multivariate analysis of variance (MANOVA; see Table 8). The only significant difference between the groups was in the familiarity ratings, with the within-subjects ratings being significantly higher than the between-subjects ratings. Given that for the within-subjects design all participants rated familiarity first, thus avoiding any of the issues associated with order effects, this result speaks to the lack of reliability found in rating studies rather than suggesting fundamental differences in the ratings, depending upon the design of the study.

**Table 8:** MANOVA results for comparisons between within-subjects and between-subjects ratings

	*F*	Sig.	Partial Eta-Squared
Familiarity	5.805	.017	.028
Meaning	0.026	.872	.000
Literality	1.197	.275	.006
Decomposability	0.528	.468	.003

Due to the difference in familiarity ratings between the groups, and Nordmann et al.’s (2014) suggestion that familiarity with an idiom impacts other ratings, a one-way multivariate analysis of covariance (MANCOVA) with familiarity entered as a covariate was also conducted upon the mean ratings for meaning, literality, and decomposability. After controlling for the effect of familiarity, we found a significant difference between the meaning ratings, but no difference for literality or decomposability (see Table 9). Given the strong correlation between familiarity and meaning, it is unsurprising that significant differences in the familiarity ratings between the groups would result in a difference in meaning ratings after controlling for familiarity. What is more interesting is the lack of differences between the ratings for literality and decomposability, regardless of the inclusion of familiarity as a covariate, given that these two variables were most susceptible to order effects in the within-subjects design. Our results suggest that the ratings that participants give are not influenced by order effects and are fixed in nature. This reinforces our conviction that although study design should be carefully considered, the only thing that has any tangible effect on ratings is familiarity. Related to the analyses conducted upon age, it would be interesting for future studies to longitudinally investigate whether individual ratings change over time or whether a participant’s initial analysis of, for instance, literality remains relatively constant across the lifespan.

**Table 9:** MANCOVA results for comparisons between within-subjects and between-subjects ratings

	*F*	Sig.	Partial Eta-Squared	Estimated Mean Within (*SE*)	Estimated Mean Between (*SE*)
Meaning	17.279	.000	.783	6.15 (0.04)	6.39 (0.04)
Literality	1.143	.286	.006	4.76 (1.33)	4.56 (1.33)
Decomposability	1.771	.185	.009	3.96 (1.12)	4.17 (1.12)

#### Relationships between the ratings

**Fig. 3:**
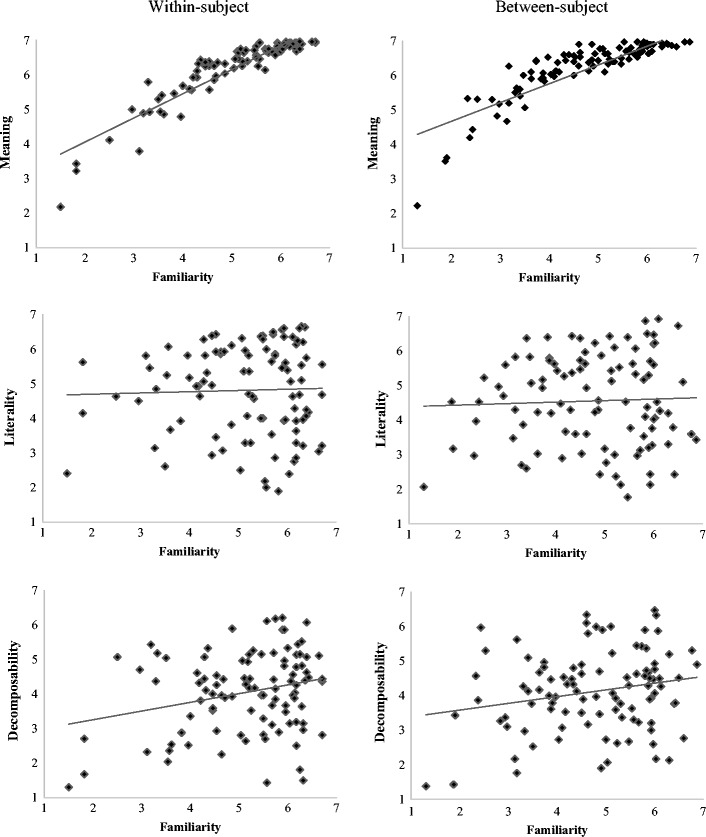
Scatterplots for correlations of familiarity with literality and decomposability

**Fig. 4:**
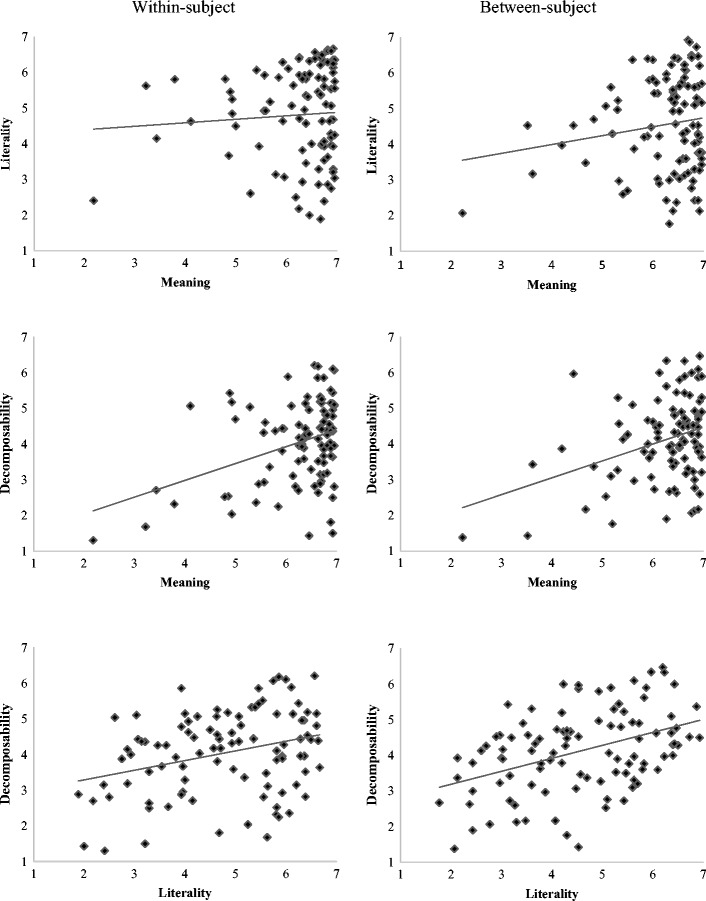
Scatterplots for correlations of meaning with literality and decomposability, and of decomposability with literality

Spearman’s Rho correlations were calculated to determine the relationships between the ratings for each design type (see Table 10, and Figs. 3 and 4 for scatterplots of the subjective measure correlations). The patterns of significance were identical for both the within-subjects and between-subjects ratings, and when the ratings from the two design types were combined. Additionally, Spearman’s Rho correlations were performed to determine the relationship between the within-subjects and between-subjects ratings for each type of rating. The ratings given from each design type were all strongly correlated (see Table 11).

**Table 10:** Spearman’s Rho correlations between the ratings by design type

	Meaning			Literality			Decomposability			Noun Frequency			Verb Frequency			Objective Freq		
	w-s	b-s	C	w-s	b-s	c	w-s	b-s	c	w-s	b-s	c	w-s	b-s	c	w-s	b-s	c
Fam.	.915^**^	.886^**^	.933^**^	.013	.007	.008	.179	.161	.156	.170	.159	.174	.200	.155	.177	.411^**^	.448^**^	.445^**^
Mean.				.05	.033	.031	.255	.222	.229	.149	.092	.125	.167	.136	.159	.349^**^	.401^**^	.400^**^
Lit.							.266^*^	.395^**^	.365^**^	–.053	–.038	–.045	–.274^*^	–.236^a^	–.255^*^	–.003	–.058	–.021
Decom.										.246	.112	.110	–.001	.017	.001	.134	.07	.089

w-s = within-subjects, b-s = between-subjects, c = combined ratings. ^*^ *p* < .01, ^**^ *p* < .001. ^a^ *p* = .018

**Table 11:** Spearman’s Rho correlations between within-subjects and between-subjects ratings

Familiarity	Meaning	Literality	Decomposability
.947^*^	.845^*^	.948^*^	.906^*^

^*^ *p* < .001

Given that idiom length was controlled for, this was not included in the analysis for Study 2; however, unlike in Study 1, we were able to include objective idiom frequency, noun frequency, and verb frequency. Frequency values were obtained from the British National Corpus (2007; henceforth, *BNC*). For objective idiom frequency values, it is likely that the BNC underestimates frequency due to lexical and syntactic variation. For example, the idiom *come to your senses* can be produced as *came to your senses*, *come to his senses*, *came to her senses*, and so forth. To try to reduce the impact this variation had on the frequency values obtained, the pronoun or article of each expression was replaced by a free operator, in the form *come to _ senses*, to allow the corpus to retrieve as many variations as possible. The frequency values produced would still be underestimated, since this search procedure does not allow for more creative idiomatic variations that would include additional lemmas (e.g., *come to your bloody senses*). Finally, 15 idioms could not be found in the corpus in any form, so the correlations performed with objective frequency have *N* = 85. As in Study 1, alpha was set at *p* < .01. For brevity, we do not report the correlations between the objective measures, but these are available at http://osf.io/igqyn.

The results of Study 2 provide strong evidence that the design of rating studies does not impact the ratings that are obtained, with no difference between the datasets (with the exception of familiarity) and both sets of ratings being strongly correlated. The relationships between the ratings replicate previous findings for familiarity and meaning. Additionally, the positive correlation with objective frequency ratings obtained from the BNC adds weight to the validity of the subjective measure of familiarity. As was stated in the Method section, it is likely that the BNC frequencies underestimate the real prevalence of each item, but it is gratifying that the relationship between subjective and objective is still present. We failed to replicate the positive correlation between familiarity and decomposability found in Study 1 and in Bonin et al. (2013), Libben and Titone (2008), and Nordmann et al. (2014). This highlights the importance of meta-analyses when drawing conclusions based on studies that use different items from different languages, which is the focus of Study 3.

## Study 3

### Method

The relationships reported from six previously published studies were included in a meta-analysis: Bonin et al. (2013), Caillies (2009), Libben and Titone (2008), Nordmann et al. (2014), Tabossi et al. (2011), and Titone and Connine (1994). In addition, the three sets of results from the present article were included (Study 1, Bulgarian; Study 1, English; Study 2, combined between- and within-subjects results), for a total of nine datasets in the meta-analysis. Cronk et al. (1993) was not included in the meta-analysis, since their studies only provide normative ratings for familiarity and literality. More importantly, the instructions given to participants for the literality judgments differed markedly from those in other studies. Cronk et al. asked participants to rate how often they had heard the idiom used literally, rather than whether the idiom has a permissible literal interpretation. Additionally, Gibbs and Nayak (1989) and Gibbs, Nayak, Bolton, and Keppel (1989) asked participants to categorize idioms as nondecomposable, decomposable, and abnormally decomposable, and the items used were preselected based on the authors’ intuitions that an equal number of each fell into each group. Due to the use of a different scale and the preselection of items, these studies were not included in the meta-analysis.

The included normative studies did not all measure the same variables. Only those relationships that were measured in a minimum of two studies (see Table 12) were considered, so some additional variables, such as syntactic flexibility (Tabossi et al., 2011) and cumulative frequency (Bonin et al., 2013), that have previously been investigated are not included in the meta-analysis. Titone and Connine (1994) reported values for nondecomposable, normally decomposable, and abnormally decomposable ratings. It was unclear which rating best mapped on to the scales used by other studies, and for this reason the decomposability ratings from Titone and Connine were not included in the meta-analysis. Libben and Titone (2008) also used the same procedure as Titone and Connine, but they reported a combined decomposability rating and were therefore included in the analysis.

**Table 12:** Correlations provided by each normative study

	Fam.	Mean/Knowledge	Lit.	Predict.	Decom.	Noun Freq.	AoA	Verb Freq.	Length
Bonin et al.	✓	✓	✓	✓	✓	✓	✓	✓	✓
Caillies	✓	✓	✓	✓	✓				
Libben & Titone	✓	✓	✓	✓	✓	✓		✓	
Nordmann et al.	✓	✓	✓		✓				
Nordmann & Jambazova (Exp. 1: Bul)	✓	✓	✓		✓				✓
Nordmann & Jambazova (Exp. 1: Eng)	✓	✓	✓		✓				✓
Nordmann & Jambazova (Exp. 2)	✓	✓	✓		✓	✓		✓	
Tabossi et al.	✓	✓	✓	✓	✓		✓		✓
Titone & Connine	✓	✓	✓	✓	!				

Fam = familiarity, mean = meaning, lit = literality, predict = predictability, freq = frequency, ! = ratings conducted categorically.

Libben and Titone (2008), Nordmann et al. (2014), and the analyses presented in the present article were all based on Spearman’s Rho correlations, whereas the remaining studies used Pearson’s correlations. Koricheva, Gurevitch, and Mengersen (2013, p. 201) stated that for meta-analyses, when *N* >̲ 90, *r* = *r*_*s*_, and therefore the values were included as reported rather than being transformed. The final difference between the included studies was the use of different rating scales for familiarity, meaning, literality, and decomposability ratings. Seven-point Likert scales were used in the present article, Nordmann et al. (2014), and Tabossi et al. (2011); five-point Likert scales were used in Bonin et al. (2013) and Libben and Titone (with the exception of the decomposability ratings, which were categorical as noted above); and Caillies (2009) used a six-point Likert scale. Despite differences in the rating scales, all reported correlations were included in the analysis. Although the summary statistics for the ratings are not comparable without prior transformation, our meta-analysis was concerned with the correlations between the ratings rather than the ratings themselves. Additionally, Colman, Norris, and Preston (1997) found a very strong correlation (*r* = .92) between 5-point and 7-point Likert scale responses.

### Results

**Fig. 5:**
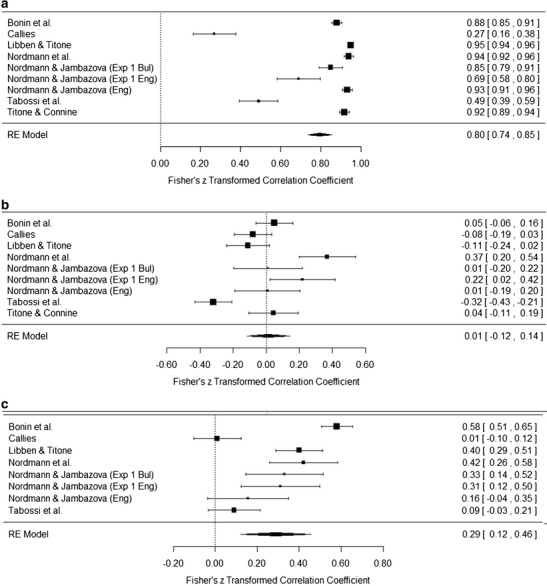

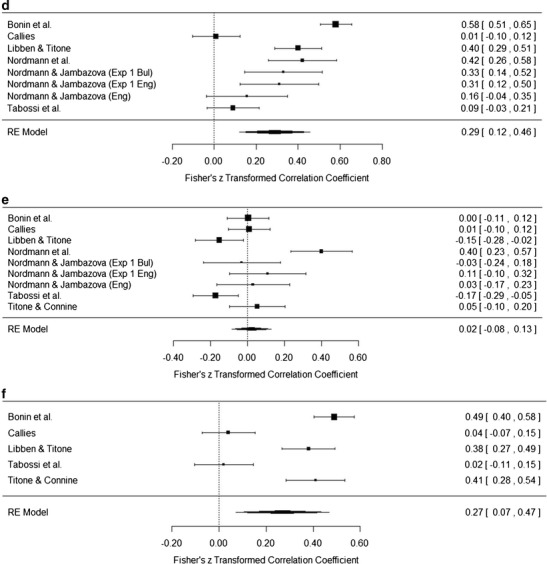

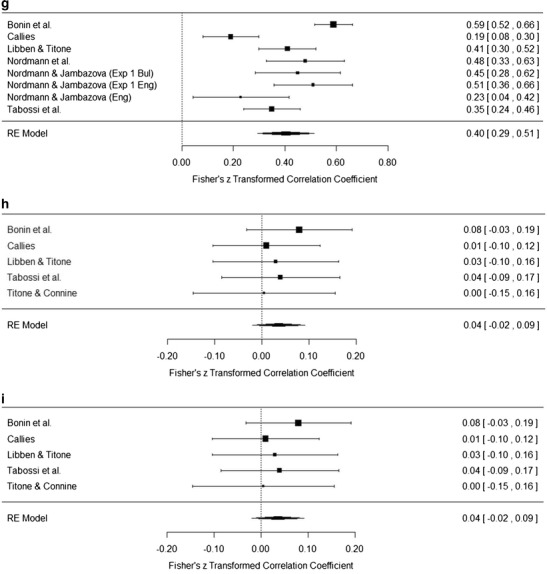
**a** Forest plot for the familiarity–meaning meta-analysis. **b** Forest plot for the familiarity–literality meta-analysis. **c** Forest plot for the familiarity–predictability meta-analysis. **d** Forest plot for the familiarity–decomposability meta-analysis. **e** Forest plot for the meaning–literality meta-analysis. **f** Forest plot for the meaning–predictability meta-analysis. **g** Forest plot for the meaning–decomposability meta-analysis. **h** Forest plot for the literality–predictability meta-analysis. **i** Forest plot for the literality–decomposability meta-analysis

The meta-analysis was conducted using the DerSimonian–Laird random-effect meta-analytical approach with correlation coefficients as effect sizes, as described by Schulze (2004), using the metafor package (Viechtbauer, 2010) in R (R Development Core Team, 2012). Table 13 presents the results of the meta-analysis, including file drawer *N* calculated using the Rosenthal method (Rosenthal, 1979). Figures 5a–i present forest plots for the meta-analyses. We only present plots for those analyses that included the data from five or more studies; the full set of plots is available at http://osf.io/igqyn. As in Studies 1 and 2, alpha was set at *p* < .01.

**Table 13:** Meta-analysis results

	Meaning/Knowledge	Literality	Predictability	Decomposability	Noun Frequency	Verb Frequency	AoA	Length
Familiarity	.80^**^	.01	.34^**^	.29^**^	.25^**^	.09	–.66^**^	–.03
	.75, .85	–.12, .14	.20, .48	.12, .46	.19, .32	–.14, .31	–.81, −.51	–.10, .04
	*N* = 9	*N* = 9	*N* = 5	*N* = 8	*N* = 3	*N* = 3	*N* = 2	*N* = 4
	91,082		377	505	42		617	
Meaning/knowledge		.02	.27^*^	.40^**^	.21^**^	.05	–.47^*^	.05
		–.08, .13	.07, .47	.29, .51	.13, .29	–.14, .24	–.82, −.12	–.14, .24
		*N* = 9	*N* = 5	*N* = 8	*N* = 3	*N* = 3	*N* = 2	*N* = 3
			231	1,036	26		222	
Literality			.04	.12	.05	–.02	.04	–.11
			–.02, .09	–.07, .32	–.03, .13	–.20, .17	–.26, .33	–.29, −.08
			*N* = 5	*N* = 8	*N* = 3	*N* = 3	*N* = 2	*N* = 3
Predictability				.16	.23^**^	–.14^**^	.16	*N*/A
				.02, .30	.13, .32	–.25, −.03	.26, .58	*N*/A
				*N* = 4	*N* = 2	*N* = 2	*N* = 2	*N* = 1
					20	7		
Decomposability					.24^**^	–.06	–.27	.16
					.10, .38	–.18, .05	–.59, .04	.03, .29
					*N* = 3	*N* = 3	*N* = 2	*N* = 3
					44			

Mean *r* values, 95 % confidence intervals of the effect sizes, numbers of studies included in the analysis, and file drawer *N* for the significant analyses are reported on separate lines. AoA, age of acquisition. ^*^ *p* < .01, ^**^ *p* < .001

Broadly speaking, the results of the meta-analyses can be grouped into four sets of interest, on the basis of the strength of the correlations and the variables involved. Rosenthal (1979) suggested a file drawer tolerance level of five times the number of studies included in the meta-analysis + 10. Using this criterion, those results with problematic file drawer *N*s were also those with weak correlations.

Familiarity and meaning are both strongly correlated with each other and with age of acquisition (AoA; *r* ≥ .5) whereas familiarity and predictability are moderately correlated, and these form a “subjective knowledge” set of correlations. This set of correlations is not particularly surprising, since it mirrors the findings of all individual studies and has a logical root: How well we know an idiom is related to when we first encountered the idiom and to its subsequent frequency in our everyday language experience.

The second set, of moderate correlations (*r* = .3–.49), is theoretically more interesting. Decomposability positively correlates with familiarity and meaning, suggesting a reliable relationship across languages between measures of subjective frequency and whether participants view an idiom as semantically decomposable. This set of correlations supports previous arguments by Keysar and Bly (1995) and Nordmann et al. (2014) that once a speaker knows the meaning of an idiom, they essentially lose the ability to objectively decide whether the figurative meaning is compositional. These relationships may also explain some of the inconsistencies in experimental studies that have manipulated decomposability. For example, in post-hoc analyses Sprenger, Levelt, and Kempen (2006) found that the priming effects they observed for idiom production were not influenced by decomposability, although there was some sensitivity to decomposability for speech onset latencies. In a study of tip-of-the-tongue (TOT) states for idiomatic expressions, Nordmann, Cleland, and Bull (2013) found no effect of decomposability upon TOT incidence, but they did find that participants were more likely to recall a greater proportion of words for a decomposable than for a nondecomposable idiom while in a TOT. In a comprehension study, Caillies and Butcher (2007) found faster processing for decomposable idioms preceded by a neutral context for a lexical-decision task, with participants being faster to respond to decomposable than nondecomposable idioms. Cutting and Bock (1997), on the other hand, found no effect of decomposability in a production study looking at idiom blends in experimentally elicited speech errors.

It is possible that these conflicting findings are a result of the different tasks that each experiment administered, with conflicts arising from different uses of context, judgments, and comprehension versus production. However it is also possible that these differences are a result of the confounding effect of familiarity. We know that there is great variability in the familiarity ratings given to specific idioms, from the reliability analyses conducted in the present article and in Nordmann et al. (2014). It follows that categorizing experimental stimuli into high- and low-familiarity or -decomposability groups on the basis of normative data rather than the ratings of the speakers who participate in the experimental studies is problematic (although this is not to say that normative datasets are not very useful). We raised this issue in Nordmann et al., and the results of the meta-analysis only strengthened our argument. In order to get a true account of the effect of decomposability (or any other feature) in experimental work, it is essential that the idioms used also be rated by the people who participate in the experiments, so that any idioms that are unknown to individual participants can be removed from the analysis. The nuances of what constitutes an idiom, a proverb, or a fixed expression can be debated, but at the most basic level what defines these types of expressions is that they are not novel, they are *known*. It is possible that the inconsistencies found in experimental work are due to the inclusion of items that are not in fact idioms, in the known sense of the definition, for individual participants.

The third set of significant correlations are weak (*r* = .1–.29) and predominantly involve the objective measures of noun frequency, verb frequency, and length. Given that these correlations are based on findings from only two or three studies and have small file drawer *N*s, we are reticent about drawing strong conclusions at this point (it should also be highlighted that the AoA meta-analyses are also based on the results of two studies). The results of the meta-analyses largely agree with those of Libben and Titone (2008): Noun frequency is correlated with all measures except literality, but verb frequency is only negatively correlated with predictability, and Libben and Titone attributed this to the stronger semantic role that nouns play than verbs. Meaning was also weakly correlated with predictability, a relationship we suggest has roots in the link with familiarity.

The final set that is of interest is the lack of correlations between literality and any other variable. In Nordmann et al. (2014), we found positive correlations for literality with both familiarity and decomposability, and argued that idioms that have a permissible literal interpretation may be easier to learn and are seen as more semantically decomposable, due to the difficulty of distinguishing literal and nonliteral meanings (Ariel, 2002). In contrast, the conflict between literal and figurative meanings as a unique property of idioms has been used to explain Libben and Titone’s (2008) findings of a negative correlation with decomposability, and also Tabossi et al.’s (2011) findings of a negative correlation with familiarity. Our meta-analysis suggests that all of these arguments are unnecessary, since it is likely that these findings were item-specific, and when taking a broad view of idiomatic expressions, literality does not have any relationship with any other subjective rating.

## Discussion

The present article had three main aims: to provide normative data for a set of Bulgarian idioms and their translations, to empirically investigate whether the type of research design employed in normative studies affects the data collected and the conclusions drawn, and to conduct a meta-analysis allowing a broader view of the relationships between subjective ratings of idiomatic expressions.

Study 1 not only provides a resource of normative data for translated idioms, but for the first time extends research into the relationships between the subjective ratings of idioms to a Slavic language. Bulgarian has a complex system of verbal conjugations that are markers for person, number, and tense of the verb. Verb forms, in turn, agree with the subject in terms of person, number, and gender. Unlike in English, in Bulgarian there is relatively free word order in sentences (Stamenov & Andonova, 1998), and sentence processing relies more on semantic–pragmatic information (Haarmann, Cameron, & Ruchkin, 2003). That the relationships between the ratings for the Bulgarian idioms largely mirror those found in other languages, such as French (Bonin et al., 2013), English (Libben & Titone, 2008; Nordmann et al., 2014; Titone & Connine, 1994), and Italian (Tabossi et al., 2011) adds weight to the argument that these relationships can be generalized to idioms as a universal feature of language, rather than being language-specific.

The results of Study 2 indicate that the ratings obtained from between- and within-subjects designs are comparable and that any differences in the ratings do not affect the pattern of correlations between the ratings. We still argue that a within-subjects design is preferable, in recognition of the fact that these subjective ratings are not independent and should not be treated as such. However, in reality the design appears to have a negligible effect. The choice of which design to use may come down to whether one is interested in the characteristics of idiomatic expressions as linguistic features, or whether one wishes to study how speakers of a language perceive these characteristics—for the former question, a between-subjects design may be preferable; for the latter, within-subjects.

Finally, the meta-analysis has brought together numerous normative studies and clarified some of the more controversial relationships between the subjective ratings. The inconsistent results surrounding decomposability reported in individual studies have stabilized into reliably moderate positive correlations between decomposability ratings and measures of subjective frequency. This finding should be taken into account when selecting experimental stimuli. The inconsistencies surrounding literality have also stabilized into a clear lack of correlations. We are confident enough in the results of the meta-analysis to go against our previous findings (Nordmann et al., 2014) and to state that there is very little evidence that literality ratings have any relationship with other subjective measures. Regarding the correlations between the subjective and objective measures, we found some evidence that the objective frequency of the component words, particularly nouns, plays a role similar to that of familiarity in how idioms are perceived, and this is supported by the moderate significant correlation in Study 2 between familiarity and objective BNC frequency. However, more data need to be collected before firm conclusions can be drawn regarding the objective measures.

## Footnotes

1 We also thank an anonymous reviewer of an earlier version of this article for highlighting the importance of this issue.
